# Viral infectious diseases severity: co-presence of transcriptionally active microbes (TAMs) can play an integral role for disease severity

**DOI:** 10.3389/fimmu.2022.1056036

**Published:** 2022-12-02

**Authors:** Aanchal Yadav, Rajesh Pandey

**Affiliations:** ^1^ Division of Immunology and Infectious Disease Biology, INtegrative GENomics of HOst-PathogEn (INGEN-HOPE) laboratory, CSIR-Institute of Genomics and Integrative Biology (CSIR-IGIB), Delhi, India; ^2^ Academy of Scientific and Innovative Research (AcSIR), Ghaziabad, India

**Keywords:** infectious diseases, transcriptionally active microbes (TAMs), disease severity, immune response, RNA viruses, microbiome, microbial biomarkers

## Abstract

Humans have been challenged by infectious diseases for all of their recorded history, and are continually being affected even today. Next-generation sequencing (NGS) has enabled identification of, i) culture independent microbes, ii) emerging disease-causing pathogens, and iii) understanding of the genome architecture. This, in turn, has highlighted that pathogen/s are not a monolith, and thereby allowing for the differentiation of the wide-ranging disease symptoms, albeit infected by a primary pathogen. The conventional *‘one disease - one pathogen’* paradigm has been positively revisited by considering limited yet important evidence of the co-presence of multiple transcriptionally active microbes (TAMs), potential pathogens, in various infectious diseases, including the COVID-19 pandemic. The ubiquitous microbiota presence inside humans gives reason to hypothesize that the microbiome, especially TAMs, contributes to disease etiology. Herein, we discuss current evidence and inferences on the co-infecting microbes particularly in the diseases caused by the RNA viruses - Influenza, Dengue, and the SARS-CoV-2. We have highlighted that the specific alterations in the microbial taxonomic abundances (dysbiosis) is functionally connected to the exposure of primary infecting pathogen/s. The microbial presence is intertwined with the differential host immune response modulating differential disease trajectories. The microbiota-host interactions have been shown to modulate the host immune responses to Influenza and SARS-CoV-2 infection, wherein the active commensal microbes are involved in the generation of virus-specific CD4 and CD8 T-cells following the influenza virus infection. Furthermore, COVID-19 dysbiosis causes an increase in inflammatory cytokines such as IL-6, TNF-α, and IL-1β, which might be one of the important predisposing factors for severe infection. Through this article, we aim to provide a comprehensive view of functional microbiomes that can have a significant regulatory impact on predicting disease severity (mild, moderate and severe), as well as clinical outcome (survival and mortality). This can offer fresh perspectives on the novel microbial biomarkers for stratifying patients for severe disease symptoms, disease prevention and augmenting treatment regimens.

## Introduction

The resident microorganisms inside the host body, collectively known as commensal microbiota, have been investigated for their functional role in modulating the human health in a plentitude of studies. This has been expedited and expanded by the global consortium efforts, inclusive of Human Microbiome Project (HMP) (1) and METAgenomics of the Human Intestinal Tract (MetaHIT) (2). At the same time, these multi-pronged multi-partner initiatives highlighted the spatio-temporal variability/dynamics as well as functionality of the microbial community. During this process, the tools for experiments, analysis, inferences and functional elucidation have strengthened the microbial genomics field (3). Thanks to their interactions within the human body that contribute towards many vital functions, they have also been referred to as our *forgotten organ* (4). The commensal microbiota influences various aspects of host physiology, including the immune development, homeostasis and functionality (5). Both the innate and adaptive immune systems are impacted by interactions between the host and microbes. Studies showing that commensal microbiota have direct and indirect effects on monocytes, macrophages, lymphoid cells, B cells, regulatory T cells, and dendritic cells (DCs) have highlighted a thorough hierarchical functionality of this interaction (6).

**Figure 1:**
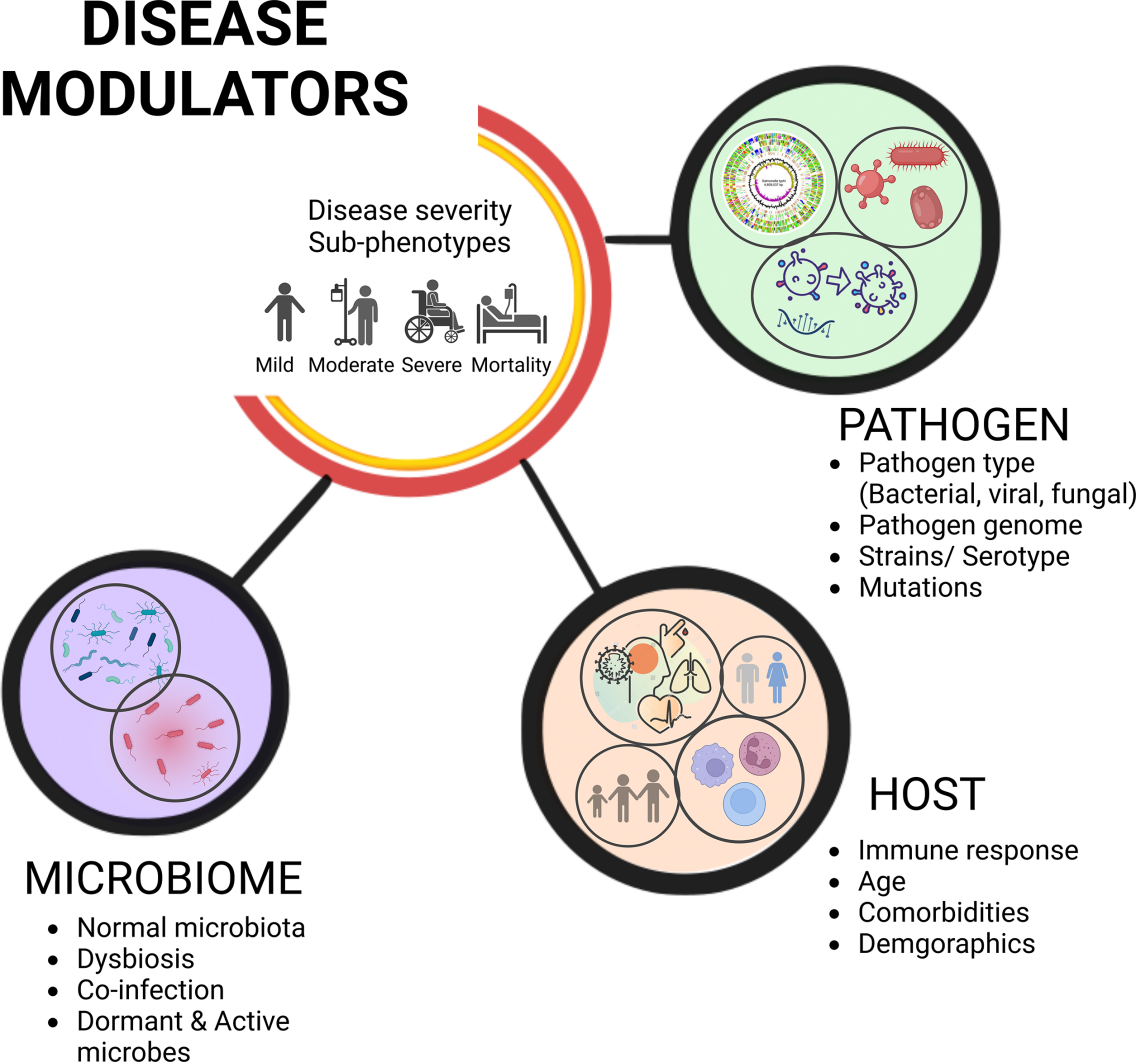
Disease severity sub-phenotypes, clinical outcome, and modulators. The complex yet intricately regulated interplay of variables governs disease severity sub-phenotypes and differential clinical outcome. Understanding pathogen and host responses integrally, along with the role of microbes, influence the trajectories of disease progression and clinical outcome.

Despite the significant roles played by the commensal microbes, when a pathogen gains access to the host, we often focus on understanding the host-pathogen interactions, overlooking the functional influence of microbiota on the infection’s severity and the clinical outcome. A systemic approach to infectious diseases integrates the pathogen genomic components, host defence mechanisms against the pathogen, and knowledge of the human microbiome, which all work together to modulate the disease sub-phenotypes (7) (**Figure 1**). There is a wealth of evidence advancing our knowledge about significant interactions between these residential commensal microbes of the host and the infecting primary pathogens that cause serious health problems, including the infections caused by the viruses (8, 9). These interactions disrupt the coherence of commensal microbiota, altering the microbial population (termed as dysbiosis) thereby, either benefitting the host or fostering the pathogen proliferation resulting in a relatively severe infection (10). Consequently, commensal organisms may switch over to potential/opportunistic pathogens and cause a secondary infection to the host. However, the impact of this subsequent secondary infection depends on the active members of the microbial community at a given point of time, or during a particular disease state; the transcriptionally active ones may have the potential to alter the disease state of a primary pathogen infected individual/s (11). In order to characterize systematically the transcriptionally active microbiota, high-throughput sequence-based studies must be conducted, as only a subset of microbes can be cultured for understanding their genome architecture and functional metagenomics-based mechanistic elucidation. Next generation sequencing (NGS) (culture-independent methods) can complement the culture-based methods, because of the limitations associated with culturing the slow-growing and anaerobic microorganisms (12). The advancements of automated, high-throughput NGS platforms allows for culture-independent analyses that can readily identify a large proportion of the microbial diversity that can be difficult to observe with culture-based studies. Most microbial sequencing techniques, including the 16S rRNA gene sequencing and shotgun metagenomic sequencing, sequence DNA extracted from samples that can be active, inactive, or dead microbes (13). As a result, targeted elucidation of RNA profiles using a variety of methods could aid us in identifying microorganisms that are functionally/transcriptionally active (14). This approach captures biological perspective by virtue of transcription representing both active cellular response to stimuli and active replication of the species (15). Since RNA has a shorter life span, the sequencing libraries constructed using reverse transcribed 16S rRNA as the amplification template, or meta-transcriptomics (RNA-Sequencing), have opened up the panoramic vista for the identification of multiple metabolically/transcriptionally active microbes simultaneously (16, 17).

Meta-transcriptomics analysis of microbiome RNA-seq data yields precise insights of the active member of the microbiota, capturing the microbiome profiles at high resolution along with the active functional elements. Although RNA sequencing has made it possible to examine the expressed transcripts of the active members of the microbiome, it still has some experimental and analytical limitations. A significant challenge is the lack of sufficient reference genomes, resulting in an inability to accurately assign and annotate all the sequence reads to the known transcripts for a particular genome/microbe (18). This is also compounded by the fact that the complete culturable condition and functional annotation is known for a very small subset of the microbial community. Furthermore, meta-transcriptomics evaluate mRNA expression profiles in order to identify the active microbial species, but because mRNA has a short half-life, it poses a serious obstacle to detect quick and short-term responses to the environmental changes (19). Another confounding factor is related to undesired sequencing reads towards the host mRNA and rRNA which in turn increases the sequencing cost for the study for capturing the dynamics of the microbial composition.

Such RNA-based studies on the active microbiome are very limited thus far, including a recent study by our group, wherein we highlighted the significant differential presence of transcriptionally active microbial isolates as possible modulators of COVID-19 disease pathology (18). Given the paucity of evidence regarding the availability of transcriptionally active microbial isolates, we introduce the term “Transcriptionally active microbes (TAMs)”, emphasizing the importance of identifying the active microbial communities. We provide a compendium of insights on the possible roles of TAMs, which could act as potential pathogens, and also their differential abundance in clinical sub-phenotypes of a disease condition.

Notably, in real-world setting, concomitant infections predominates a single pathogen infection as observed during the coronavirus disease 2019 (COVID-19) pandemic, where limited yet significant studies demonstrated that the majority of the infected patients reported co-presence/co-infections of other microbes, in addition to the primary infecting severe acute respiratory syndrome coronavirus 2 (SARS-CoV-2) (19). This in turn has been linked to worsening of the disease outcome (20). Felman and Anderson, described the incidences of co-infections in individuals with SARS-CoV-2 infection (21). Although a myriad of published literature documented co-infections with other respiratory viruses, bacteria and fungi, the precise functions of the occurrences of different pathogens remains unclear. Consequently, understanding the co-infection timing, patterns and spectrum to evaluate the clinical features of patients is important, particularly for recurrent infectious agents that cause global health threats, such as SARS-CoV-2, Influenza, and Dengue virus. It is important to highlight that all of these are single-stranded RNA viruses responsible for significant infections worldwide, wherein co-infections with other microbes have been correlated with disease severity and mortality in a subset of the patients (22–24). Timely monitoring of the co-infecting pathogens could help better understand the role TAMs play in the pathogenesis of these viral infections. As mentioned earlier, with the use of gold standard bacterial cultures towards mechanistic understanding through functional metagenomics-based approaches, there arose diagnostic doubts due to the false negatives as well as the increased time and sample volume usage. The increased diagnostics for pathogen detection as well as co-infections has been well aided by the molecular diagnostic approaches using reverse transcription polymerase chain reaction (RT-PCR), and loop-mediated isothermal amplification (LAMP). These techniques can estimate the burdens of co-infecting pathogens, as revealed by a study wherein they found frequent *H. pylori* and *Shigella spp*, co-infection as the cause of diarrhoea using RT-PCR (25). The LAMP was developed in the year 2000 and has also been used for co-infection detection in patients with HIV-Leishmania co-infection with 100% diagnostic efficacy and specificity (26). Both have applications towards pathogen presence/absence as well as differential abundance of the specific genomic regions. This has preceded application and success of pathogen genomic surveillance, including SARS-CoV-2. They have ably and timely aided the NGS based pathogen genome information through functional role of the mutations vis-à-vis disease severity, clinical outcome, immune escape and pathogenicity. Thereby, a deeper comprehension of the prevalence of additional TAMs in these RNA virus-infected patients can augment specific functional studies, effective patient management and treatment stewardship during these disease conditions.

## Understanding the viral pathogens

Infectious diseases continue to be the single most serious threat to public health, healthcare management as well as significant impact on the global economies, accounting for the 2^nd^ leading cause of death and disability worldwide. The infectious microorganisms encompass a wide variety of organisms including not only bacteria, fungi, protozoa, worms, viruses, but also unusual infectious proteins known as prions. There exists a striking diversity amongst these pathogens. Understanding the pathogen idiosyncrasy is essential and urgent to comprehend the trajectories of disease progression and clinical outcome.

Among the spectrum of pathogens, viral pathogens, particularly RNA viruses, are considered to be at the leading edge of human pathogens, accounting for ~25%-44% of all the infectious diseases. This is often because of their relatively higher rates of nucleotide substitution, compromised mutation error-correction ability and therefore higher capacity to adapt to the new hosts (27). Due to the high prevalence of mutations in their genomes, the outcome of concomitant infections by RNA viruses might generate a more virulent strain, augmenting viral fitness. Through specific RNA viruses, Influenza, Dengue, and SARS-CoV-2 presented herein, that have impacted a substantial proportion of global population, including India and south-east Asia, home to ~9% of the world population, resulting in numerous infections every year (28–30), the article highlights the importance to understand this further.

What’s so special about critiquing the pathogens’ genome? How and why do the different strains of virus have different effects? The answer lies in the unique way in which each pathogen causes the disease. Viruses of the same species are phylogenetically divided into serotypes and clades based on their genomic sequences, and antigenic differences. The heterogeneity among these pathogens leads to differential susceptibility, immune escape potential, and with varying potential to cause infection, resulting in disparate clinical manifestations and disease outcome. As a result, it is challenging to consider any pathogen as a monolith. This has been clearly highlighted by the COVID-19 pandemic, wherein SARS-CoV-2 variants of concern, VOC (Alpha, Beta, Gamma, Delta and Omicron) characterized by distinct set of mutations leading to changes in the viral properties, and is one of the causal factors behind the diverse disease presentations (31).

Pathogens exhibit strain-level diversity and contribute to eliciting specific responses from the host cells. The myriad of pathogen genome sequencing has revealed the influenza virus diversity to be accounted for by different virus subtypes and strains leading to epidemics or pandemics (A/H3N2 and A/H1N1 subtypes) (32). Highly pathogenic influenza viruses include influenza virus A, B, and C of which major outbreaks are associated with types A and B, whereas influenza C is associated with common cold-like illnesses, principally in children. Only influenza virus A causes Flu pandemics (33). The observed differential disease conditions by the same pathogen are also true for dengue virus, with a range of symptoms. This can be attributed to the four dengue virus (DENV) serotypes (DENV-1, DENV-2, DENV-3, and DENV-4) are antigenically distinct, and are associated with different clinical manifestations (34). Thus, understanding an infectious disease requires investigating the pathogen genomic architecture in addition to other factors playing an increasingly important role in disease modulation.

## Host factors modulating infectious diseases outcome

Since the host adaptation for viral replication and transmission is indispensable for virus evolution, the host components play a key role in the infection pathogenesis and disease progression. As new infectious diseases continue to emerge, elucidating how host response influences the disease trajectory is of great importance. Notably, the interdependence of the host and the infecting pathogen could determine the disease susceptibility, severity and outcomes (35)

As observed in the COVID-19, the innate immune system functions as the first line of host defence, which is activated as the SARS-CoV-2 engages the angiotensin-converting enzyme 2 (*ACE2*) receptor to enter the cell and employs the cellular serine protease *TMPRSS2* for its spike (S) protein priming (36). Following the cell entry, the host pattern recognition receptors (PRRs), such as retinoic acid-inducible gene-I protein (*RIG-I*), melanoma differentiation-associated gene 5 (*MDA5*) and Toll-like receptor (TLR) recognize the virus, wherein the viral E protein induces *TLR-2*, which alleviates the innate immune activation in COVID-19 patients (37). SARS-CoV-2 proteins, ORF3a, M, ORF7a, and N, activates the nuclear factor kappa-light-chain-enhancer of activated B cells (NF-κB) and produce pro-inflammatory cytokines (IL-1, IL-6, IL-8, TNF-α) that further activates NF-κB by a positive feedback mechanism, resulting in uncontrolled inflammation in the COVID-19 patients (38).

A similar response is observed in Influenza infection, wherein upon Influenza A virus (IAV) entry into the host cell through the binding of its hemagglutinin (HA) protein to either α2,3- or α2,6-linked sialic acid (SA) cellular receptors on the airway or alveolar epithelium, the intracellular IAV is recognized by PRRs. The downstream signalling of PRRs results in the activation of the transcription factors - *NF-κB*, interferon regulatory factor 3 (*IRF3*), and *IRF7*, which trigger the expression of interferons (IFNs) and pro-inflammatory cytokines (*TNF, IL6, IL1β*); mediating antiviral responses (39).

On the other hand, DENV activates a distinct immune response wherein it infects the Langerhans cell, after viral entry in the cell *via* receptor binding to the DENV E glycoprotein. This triggers the innate immune system by recruiting innate immune cells, including monocytes and macrophages, which in turn lead to increased production of cytokines and chemokines and induce an antiviral state. The innate immune system then triggers the adaptive immune response, which is relatively slow and involves antibodies produced by B and T cells-mediated recognition and killing of viral infected cells (40). A robust adaptive immune response toward viral infections is elicited by the human host, which affects disease severity.

Although a severe immune response, such as a cytokine storm, affects the patients’ disease outcome, it is critical to note that the mortality rate among the people infected is lower, but still significant numbers. Could there be any other host factor other than essentially immune response? Are age, comorbidities, or gender can contribute as observed during COVID-19 pandemic? The quest towards providing answers to these questions has been undertaken by many researchers. Finding from such studies when seen in the context that a human body harbours approximately ten times the number of microbes compared to its own cells – are they integral to infectious disease trajectory? As microbiome is crucial to maintain a state of homeostasis, could an imbalance in their abundance/diversity upon infection offer beneficial strategies as well to combat the disease?

## Our microbiome

*Humans are more microbes than humans* – if that is true, then it must be integral to the infectious disease response. While recognizing the importance of host transcriptional responses, coding and noncoding, it is nevertheless impossible to obliterate the millions and trillions of microbial genes (the metagenome) and microbial cells (the microbiota) dynamically interacting with the host, accounting for ~90% of the total cells in the human body (41). Launched in 2007, the Human Microbiome Project has improved our fundamental understanding of the human microbiome and its contribution in normal physiology as well as effect of the microbiota on the host disease states (1). The human microbiome comprises various bacteria, viruses, and fungi inevitably interwoven with human health and disease, which exist in dead, quiescent and active states (42). The ongoing study of the human microbiome keeps shedding light on the beneficial effects of the active microbiota on fundamental physiological and metabolic processes, where they are crucial for digestion, metabolism and healthy gut physiology (43, 44). Francesc Peris-Bondia et al. highlighted the underrepresentation of the functional (active) microbiome by describing the considerable disparity between the human gut active microbial fractions and the total microbial community (11). These potentially active commensal microbes communicate with the host’s immune system through various microbial signals that can induce pro-inflammatory cytokines from macrophages and DCs, which in turn activate T-, NKT-cells and B cells *via* peptide presentation of the commensal microbiota components, thereby triggering both innate and acquired immune responses (45).

Now that we can track various microbes’ genome sequences accurately, we understand that microbiota-host interaction is mediated by several primary and secondary metabolites produced by the commensal microbial agents, the most abundant of which are short-chain fatty acids (SCFAs), mainly acetate, butyrate, and propionate (46). SCFAs mediate diverse effects on host metabolism, *via* their participation in a multitude of body functions, including the regulation of immune and inflammatory responses (47). SCFAs are the main metabolites of the metabolically active gut microbiota and an alteration in the gut microbiota’s composition is associated with decreased intestinal concentration of SCFAs. Besides, the most intriguing role of SCFA is their importance in maintaining epithelial integrity, modulating the dissemination of gut commensals, opportunistic pathogens, and microbial components. The SCFA compounds tend to reinforce the gut barrier, and hence their reduction leads to impaired barrier functions and thereby favour secondary bacterial infections (46). Thus, gut microbiota dysbiosis wherein the loss of beneficial members of the microbial community (*Lachnospiraceae* and *Lactobacillus*), and the overgrowth of pathosymbionts (*Alphaproteobacteria, Gammaproteobacteria, Escherichia* genus) leads to a barrier disruption *via* reduced SCFAs and enhanced production of toxic metabolites, resulting in microbial co-infections (48). The indigenous active microorganisms tend to inhibit the growth of potential pathogens, *via* mechanisms wherein they modulate host response-affecting infection outcome (49).

Growing evidence suggests the importance of microbiomes, which are associated with life quality and longevity. But what happens when these microbes start co-infecting or are co-present within the host along with the primary infection causing pathogen? Does the co-presence of these microbes, especially the active ones, offer benefits against the primary pathogen leading to increased survival or does it make us more susceptible to the infection by increasing disease severity and enhanced mortality rate? Can this be used as a biomarker for disease prognosis and severity prediction at the initial stages of the disease? This is a knotty issue that can be surprisingly complex and needs more spotlight.

## Connecting microbiota to infectious diseases

It has long been recognized that host health is linked to its microbial inhabitants, and disruption of the normal microbiota is associated with multitudinous health conditions. This includes allergies, autoimmune diseases, obesity, diabetes, dental diseases, brain-related disorders, inflammatory bowel disease, and respiratory illnesses, as captured by the Disbiome database, which highlights microbial composition changes in different disease states (https://disbiome.ugent.be) (50). The emerging infectious diseases share an intimate relationship with dysbiosis of the microbiota (**Table 1**), which determines the disease outcome. The oral and gut flora indicates their health status, resulting in either disease recovery or increased severity. Although the exact association between disease severity and the transcriptionally active human microbiome remains subject of focussed attention and targeted studies, the COVID-19 has revealed profound alterations in the oral, lung, gut and fecal microbiome. This indicates an abundance of opportunistic pathogens (*Haemophilus parainfluenzae, Clostridium hathewayi*) and depletion of favourable commensals (*Neisseria, Faecalibacterium prausnitzii*), which may lead to disease deterioration in the SARS-CoV-2 infected patients (52, 65).

**Table 1:** Microbiota associated with various infectious diseases.

Infectious Disease	Microbial community composition	References
COVID-19	*↓ Neisseria, Corynebacterium, Faecalibacterium prausnitzii.* *↑ Achromobacter xylosoxidans, Bacillus cereus, Streptococcus infantis, Staphylococcus epidermidis, Haemophilus parainfluenzae.*	(18, 51, 52),
Viral hepatitis	*↓ Bacteroidetes, Leptotrichia, Prevotella, Capnocytophaga, Corynebacterium, Campylobacter.* *↑ Candida, Firmicutes, Fusobacterium, Prevotella, Faecalibacterium.*	(53, 54),
Influenza	*↓ Faecalibacterium_prausnitzii, Staphylococcus aureus.* *↑ Prevotella copri, Streptococcus* spp.*, Prevotella* spp.*, Fusobacterium, Haemophilus.*	(55, 56),
HIV	*↓ Bacteroides, Alistipes.* *↑ Prevotella, Barnesiella, Candida albicans, Pseudomonas aeruginosa.*	(57, 58)
Tuberculosis	*↓ Prevotella, Lachnospira, Pelomonas aquatica, Haemophilus parahaemolyticus, Bifidobacterium, Bacteroides.* *↑ Escherichia, Streptococcus, Rummeliibacillus* sp.*, Deinococcus phoenicis, Enterococcus.*	(59, 60),
HPV	*↓ Lactobacillus gasseri, Fusobacterium nucleatum, Shuttleworthia, Lactobacillus iners.* *↑ Gammaproteobacteria, Proteobacteria.*	(61, 62)
Cholera	*↓ Bifidobacterium, Bacteroides.* *↑ Ruminococcus obeum, Escherichia coli, Enterococcus, and Veillonella.*	(63, 64)

The commensal and probiotic microbial strains provide protection against invading pathogens through various mechanisms. The tight junction structure, a crucial element that hosts use to mitigate infections, is regulated by the commensal flora, which contributes to the maintenance and improvement of the epithelial barrier integrity (66). Microbiota alteration is associated with the disruption of tight junction, with certain bacteria like *Clostridium difficile, Salmonella* and *Yersinia* spp. release toxins that impair the effectiveness of the barrier (67, 68). In addition to the release of specific inhibitory molecules by the commensal microorganisms that directly act on the pathogens, there is yet another mechanism followed by the commensal flora to limit pathogen invasion that includes the activation of host immune response (69, 70). A wealth of emerging evidence highlights the involvement of microbiota in the regulation of immune responses against infections, wherein the commensal bacteria stimulate the host’s innate and adaptive immunity to fight against pathogens. Studies in germ free or antibiotic-treated mice showed that the loss of commensal bacteria-derived signalling led to severely compromised antiviral immunity (71, 72). These findings demonstrate that the mice with altered commensal bacterial communities have an impaired type I and type II IFNs responses, which impair their ability to control infection.

These studies indicate that our understanding of the symbiotic or pathogenic microbiota and the hosts’ interaction has profoundly increased. However, extensive and specific research is still required to better understand the dynamics of the active microbial community during an infection. Bridging these gaps will pave the way not only for designing better and effective disease management strategies but also for developing novel therapeutics.

## Co-infection dynamics: causal or participant?

Despite significant progress in their prevention, most infectious diseases are difficult to eliminate and continue to pose a great threat to global health. Any infectious disease has traditionally been approached with the premise that it is caused by a single pathogen. Recent research has strengthened an alternative understanding of the presence of more than one pathogen in a substantial number of patients. Co-infecting pathogens are a key cause of mortality and morbidity in various illnesses, and modulate the disease severity trajectory, giving rise to different clinical phenotypes for a disease, ranging from mild symptoms to patients exhibiting critical illness (73). With the invasion of a pathogen, a microbiome disturbance ensues wherein a possible shift of the microbial sub-population from commensal to pathogenic may occur (74). This results in the deterioration of the human health outcomes due to the consortium of pathogenic microbes together with the primary infecting pathogen. Majority of the studies focus on the total microbial population present within the human host; however, very little is known about the active microbiota. Here we discuss the importance of understanding the metabolically/transcriptionally active ones using the RNA-Seq based approaches. Because the functionally active flora is stable over time, it is actually the one that co-infects along with the primary pathogen (75). Thus, rather than the conventional total microbiome, which includes the non-active fraction of microbes, we hypothesize that TAMs may be the major modulators of disease severity. We are now beginning to understand the interactions between the pathogens and the host’s active microbiota, which characterize how the immune system responds, thereby modulating the disease trajectory. Identifying the TAMs that act as a negative (opportunistic pathogens) or a positive (commensal microbes) regulator upon pathogenic infections is therefore critical.

Differential abundance of co-infecting pathogens has been discovered and elucidated in clinically distinct subgroups of patients, including COVID-19, where patients who require respiratory support manifest different active bacterial species than those who do not (76). Likewise, patients with community-acquired bacterial co-infections had a greater ratio of septic shock, invasive mechanical ventilation, and ICU necessity to patients without co-infections, in the influenza patients (77). Contrariwise, no difference in the disease severity among children infected with rhinovirus/enterovirus (HRV/ENT) alone compared with those co-infected with HRV/ENT and at least another virus, was observed. They in fact highlighted that a single infection leads to more severe disease compared to co-infection (78). Thus, the understanding of the overall functional importance of the co-infection in the patients deserves focussed attention to augment the hierarchical understanding of factors modulating disease severity.

There is a dearth of studies in this field, assuming the co-presence or co-infection to be always causing a severe condition is still a conundrum. Co-infections may play a pivotal role in reducing or augmenting disease severity. The simultaneous circulation of multiple potential pathogens is a serious concern that can raise the risk of co-infections. When two or more pathogens have the same seasonal pattern or overlap, they are more likely to infect simultaneously, complicating the disease pathophysiology. Co-infection may also modify the clinical presentation of a particular disease, resulting in missed or delayed diagnosis (79). It is therefore necessary to investigate the clinical outcomes of these co-infections.

## Influenza and microbiota

### If grippe condemns, the secondary infections execute, Louis Cruveilhier, 1919

As per Johnson and Mueller’s analysis of the 1918 influenza pandemic, there were ~500 million infections and 50 million deaths (80), and the disease continues to cause widespread annual epidemics, with a substantial burden of deaths globally (81). The preponderance of data points to viral and bacterial co-infection as the primary cause of most influenza cases rather than the virus itself, of which 20-30% cases are associated with severe infections, resulting in an increased risk of death. Subsequently, the mortality cases were attributed to bacterial pneumonia predominantly with *Streptococcus pyogenes, Staphylococcus aureus, Streptococcus pneumoniae* and *Haemophilus influenzae*, reflecting the importance of diagnosis of co-infections (82, 83). The first systematic review on the 2009 influenza pandemic studies by MacIntyre et al. identified *Streptococcus pneumoniae* as the most common secondary bacterial infection associated with the fatal and ICU admitted cases (84). Moreover, autopsy of influenza victims revealed extensive bacterial infection leading to increased fatality (85).

**Figure 2:**
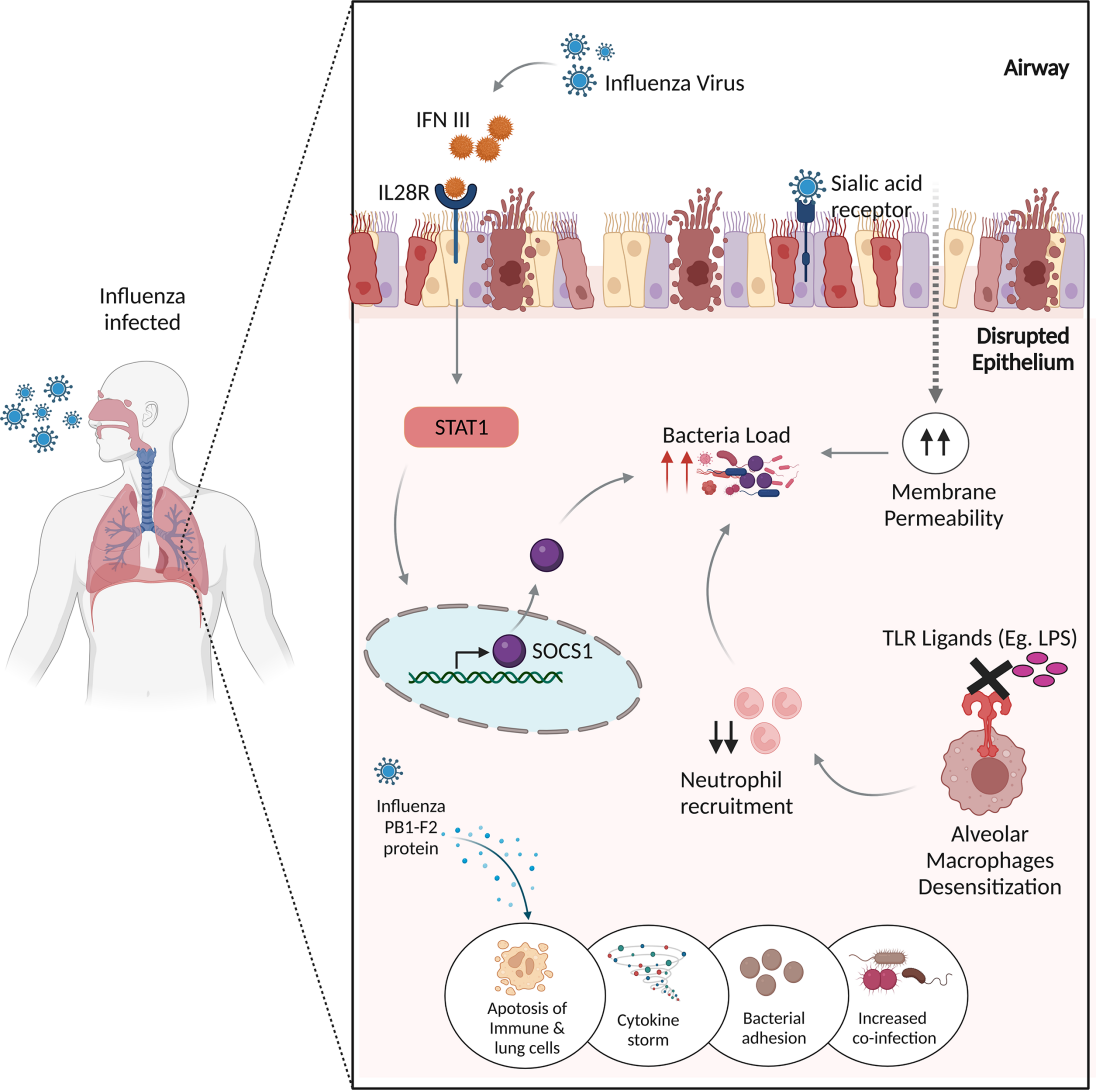
Potential mechanisms of influenza virus infection and bacterial co-infections. The influenza virus produces IFN III, which stimulates STAT1, which then activates SOCS1 to regulate antimicrobial peptides, resulting in enhanced co-infection. The binding of influenza virus to sialic acid receptors causes lung epithelial damage, increasing membrane permeability to bacterial infections. Desensitization of alveolar macrophages to TLR ligands prevents the immune system from recruiting appropriate neutrophils, resulting in increased bacterial burden. The influenza virus’s non-structural PB1-F2 protein increases inflammatory responses to co-pathogens by inducing apoptosis in immunological and lung epithelial cells.

Since bacterial co-infection has been a significant contributor to morbidity and mortality resulting in severe pneumonia, the mechanisms underlying the IAV-bacterial co-infections, are assigned to the following categories (**Figure 2**).

### Lung epithelial damage

As discussed, IAV enters the host cells *via* SA cellular receptors, therein it replicates in the epithelial cells throughout the upper and lower respiratory tract, inducing the innate immune response, which leads to direct damage of the airway epithelium (86). The viral-induced damage to the lung epithelial barrier breaches natural barriers to infection, thereby allowing the colonization of bacteria, promoting bacterial co-infection. Bacteria may take advantage of the dysfunction of lung physiology and can invade the tissues and exacerbate the disease.

### Immune response suppression

Influenza viruses modulate the immune response and inflammatory pathways, thereby generating a cytokine response and triggering an influx of immune effector cells (87). This results in manipulation of the lung immune response to the bacterial invaders by the following mechanisms.

#### ● Sustained desensitization of toll-like receptor ligand

Studies have demonstrated a protective role of TLR activation in inducing antimicrobial activity by mechanisms, including the activation of NF-κB and MAP kinase pathways and rapid acidification of the phagosome, thus eliminating or neutralizing the bacteria (88, 89). Didierlaurent et al. reported TLR dysfunction, demonstrating that the administration of flagellin, lipopolysaccharides (LPS), and lipoteichoic acid to “post-influenza” mice blocks TLR ligation, which impaired NF-κB activation and negatively affected neutrophil recruitment. This involves long-term desensitization of pattern recognition receptors (PRRs) present on the lung alveolar macrophages, which leads to a higher and prolonged bacterial load thereby increasing the susceptibility to common secondary bacterial infections (90).

#### ● Influenza virus mediated alveolar macrophage depletion

Alveolar macrophages (AMφ) are well equipped to phagocytose and kill the bacteria controlling bacterial infections. The protective role of AMφ has been demonstrated in various studies; for e.g., the mice lacking AMφ were susceptible to IAV infection and severe fatal pneumonia (91). Concurrent to this, a ferret model highlighted the significance of AMφ towards elevated levels of inflammatory chemokines in the depleted lungs infected with the H1N1 influenza virus (92). This mechanism of AMφ depletion makes the patients vulnerable to bacterial superinfections as implicated by a mice study wherein they found more than 95% AMφ-mediated bacterial clearance in mock-infected mice compared to the influenza-infected lungs (93).

#### ● Up-regulation of bacterial adhesion receptors

Influenza infection exposes the respiratory tract to increased sites for bacterial attachment. The availability of receptors allows the bacteria to enter and infect the cells (94). Various adherence molecules are expressed by these bacterias, such as choline-binding protein A (CbpA) and pneumococcal serine-rich repeat protein (PsrP) in *S. pneumoniae via* which they adhere to the membranes (95).

### Apoptosis caused by viral cytotoxin PB1-F2

When influenza attacks the infected cells, it forms a non-structural protein, called PB1-F2 that promotes inflammatory responses to co-pathogens through the induction of apoptotic death of immune and lung epithelial cells (96) (97),.

### IFN-lambda mediated increase in bacterial load

Influenza virus infection leads to the production of type III interferon, which binds to the IL-28 receptor, inducing Signal Transducer And Activator Of Transcription 1 (STAT1) phosphorylation and the expression of its regulator, suppressor of cytokine signaling 1 (SOCS1) (98). SOCS1 impacts the abundance of antimicrobial peptides in the upper airway (99). Planet et al. suggests that Il28r−/− mutant mice, which lack the receptor for type III interferon, were significantly protected from bacterial super-infections through increased production of antimicrobial peptides than the wild-type mice, which were more susceptible to methicillin-resistant *Staphylococcus aureus* (MRSA) pneumonia with an increase in the respiratory microbiota (100).

The aforementioned mechanisms help to explain why, after an infection with the influenza virus, the immune system fails to mount a successful antibacterial defence, exposing the respiratory tract to opportunistic bacterial pathogens and allowing bacterial invasion. Contrariwise, the commensal bacteria provide protection against the influenza infection by controlling the adaptive immune response, with Takeshi Ichinohe et al. discovering that an intact commensal microbiota leads to proper activation of inflammasomes. Antibiotic-treated mice had defective CD4 T-, CD8 T-, and B-cell immunity, as well as an impaired synthesis of pro–IL-1β, pro–IL-18, and NLRP3 (101). Even though its scope is constrained, the relevance of comprehensive detection of TAMs, rather than only the microorganisms, might aid to lower risk of serious flu complications, which result in 290K-650K influenza-related deaths/year worldwide (102). Thus, understanding the contribution of the active microbiota in maintaining the immunological status and rendering the immunological imbalances will ultimately allow the design of effective, broad-spectrum therapeutic approaches for prevention of enhanced susceptibility to influenza (103). Additionally, it may allow repurposing of the existing antibiotic repertoire for better clinical outcome.

## SARS-CoV-2 and microbiota

The COVID-19 pandemic brought on by SARS-CoV-2 presents a broad spectrum of severities, ranging from an asymptomatic presentation to severe pneumonia (104). These clinical sub-phenotypes may be caused by a number of variables, with co-infection being one of them. By either suppressing or priming the immune system, SARS-CoV-2 infection can change the host’s immunological response to subsequent infections, pre-infections, or co-infections by other viruses. Limited studies highlight that only a subset of patients with SARS-CoV-2 infection had microbial co-infections. Lansbury et al. highlighted the burden of bacterial co-infections in ~7% hospitalised COVID-19 patients, 14% ICU patients, 3% patients having viral co-infections, and only 3 studies reporting fungal co-infections during early COVID-19 pandemic from January-April 2020 (105). Meanwhile, another study showed that 8% of patients had bacterial/fungal co-infections (106). Both studies are cautious to note that the routine use of antibiotics in the treatment of COVID-19 infection should be judiciously investigated. Despite the paucity of investigations, it has been shown that individuals with SARS-CoV-2 infection are more susceptible when other co-infecting infections are present (107). Early studies have shown the co-infection of SARS-CoV-2 with common respiratory viruses, including rhinovirus, influenza, metapneumovirus, parainfluenza, and respiratory syncytial virus, along with bacterial species of *Staphylococcus aureus, Haemophilus influenzae*, and *Streptococcus pneumoniae* (108, 109). Even though only the active microorganisms are responsible for the biological functions, the majority of studies in COVID-19 concentrate on identifying the total microbiota rather than the active fraction (108–110). It is noteworthy that few research groups have attempted to study the transcriptionally active microbial landscape in COVID-19 patients (18, 111, 112). The study by Devi et al. identified the nasopharyngeal microbial signatures associated with the clinical sub-phenotypes of mild, moderate, severe, and mortality in the COVID-19 patients. Using holo-RNA-Seq, the significant abundance of specific TAMs was identified, with significant transcriptional presence of *Achromobacter xylosoxidans* and *Bacillus cereus* in the mortality patients, with known role in drug resistance (20).

Yang Han et al. points to SARS-CoV-2-related alterations in the active microbiota of BALF in the COVID-19 patients and healthy controls as highlighted by the significant differences of α- and β-diversity between two groups (107). Similar to this, the other metatranscriptomic study sheds light on the importance of TAMs in the COVID-19’s mild and severe patients, identifying *Burkholderia cepacia complex (BCC), Staphylococcus epidermidis*, and *Mycoplasma* spp. as the predominant respiratory active microbial taxa within the severely ill patients, whereas *Veillonella, Neisseria, Streptococcus*, and *Prevotella* in the respiratory tract of patients with mild symptoms (108).

**Figure 3:**
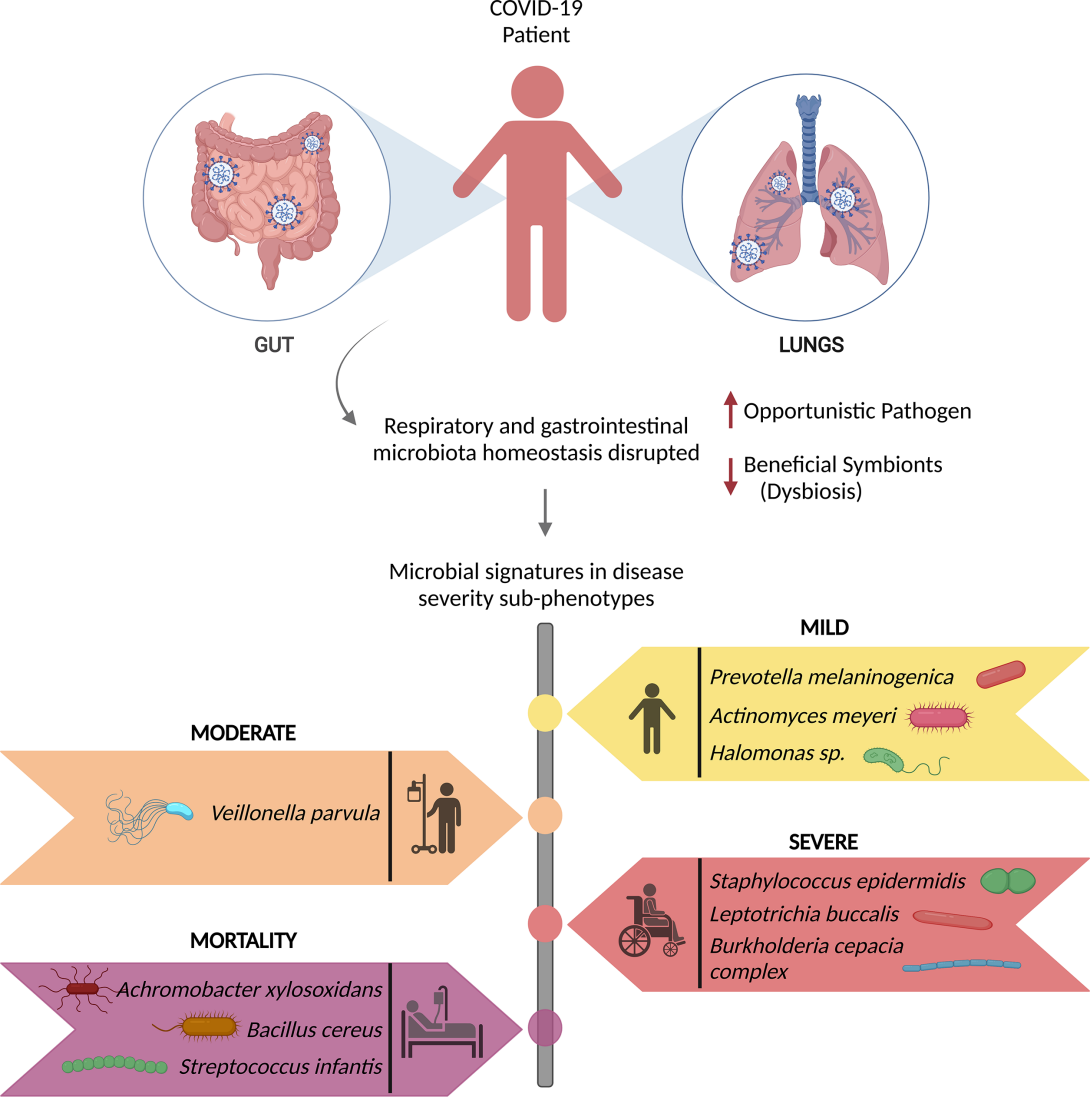
Microbial signatures associated with COVID-19 disease severity. SARS-CoV-2 infection causes gut and respiratory microbial dysbiosis, which results in several disease sub-phenotypes, mild, moderate, severe, and mortality, each with its own set of microbial signatures that can be identified as biomarkers for the specific phenotype.

SARS-CoV-2 infection has been reported to induce nasopharyngeal fungal microbiome dysbiosis with significant differences in microbiome diversity between COVID-19 patients and healthy individuals wherein *Saccharomyces cerevisiae, Candida albicans, Candida glabrata, Aspergillus flavus, Aspergillus fumigatus, Phaffia rhodozyma* and *Paecilomyces variotii* are the predominant fungal pathogens found in the infected patients (113, 114). Moreover, studies have found a significant association between the gut/intestinal microbiome alterations and disease severity in the COVID-19 patients, with commensal microorganisms being underrepresented (115, 116). The disruption of respiratory and gastrointestinal microbiota homeostasis results in persistent microbiota impairment, directly correlated with the COVID-19 severity. Recent findings have highlighted the microbial signatures for recovered (mild, moderate, and severe) and mortality patients, in **Figure 3** (18, 51).

SARS-CoV-2 infection changes the active respiratory microbiota, characterized by reduced alpha-diversity, and significantly lower microbial diversity (113). Resultantly, gut microbiota dysbiosis was observed to cause elevated levels of opportunistic pathogens, *Streptococcus*, *Rothia*, *Veillonella*, *Actinomyces* and a decreased number of beneficial symbionts, *Blautia*, *Rombontsia*, *Collinsella*, and *Bifidobacterium* (116). Along with alterations in the active microbial abundance, SARS-CoV-2 disrupts the epithelial barrier, thereby allowing the opportunistic pathogens to enter the circulation, cause systemic inflammation and thus co-infecting along with the primary SARS-CoV-2 infection. Tao et al. showed that fecal IL-18 levels positively correlated with the relative abundance of *Peptostreptococcus, Fusobacterium*, and *Citrobacter*, causing production of inflammatory cytokines in the intestine and possibly beginning of cytokine storm. Correspondingly, these changes in gut microbiota composition caused by the viral infection contribute to the severity of the disease (110).

Pathogen invasion due to microbiota dysbiosis not only facilitates the cytokine storm but also exacerbates COVID-19 because of impaired SCFAs and L-isoleucine biosynthesis derived from gut microbiota and other important gut commensal-derived metabolites/components (114). SCFA has been shown to enhance B cell metabolism in the gut, promoting anti-SARS-CoV-2 antibody production in B cells and inhibiting COVID-19 development (117). Regulatory T cells (Treg cells) are an important subpopulation of T-cells that acts as a first-line of defence against uncontrolled inflammation and viral infections by suppressing the immune system. Through SCFA and other metabolites, the commensal microbiota, directly/indirectly modulate Treg cells (118). Multiple studies have reported T lymphopenia in COVID-19 patients. Severe patients had a lower level of CD4+ and CD8+ T-cell populations than mild cases, with significantly reduced CD4+/CD8+ ratios (119, 120). Later study also demonstrated a disproportionate increase in TNF-α production and cytotoxic function from CD4+ T cells.

Not only do these microbes increase the disease severity, but they also offer some degree of protection against SARS-CoV-2, wherein patients showed significantly reduced relative abundance of *Bifidobacterium*, bacteria used in probiotics (113). By regulating crucial immunological processes, such as dampening the impact of TNF-α, and boosting the Treg responses, they augment the defence against infection (121). Towards severity prognosis of a SARS-CoV-2 infection, it may be useful to examine the composition and diversity of the active microbiome, with some microorganisms helping improve the course of the disease.

## Dengue and co-infections

**Figure 4:**
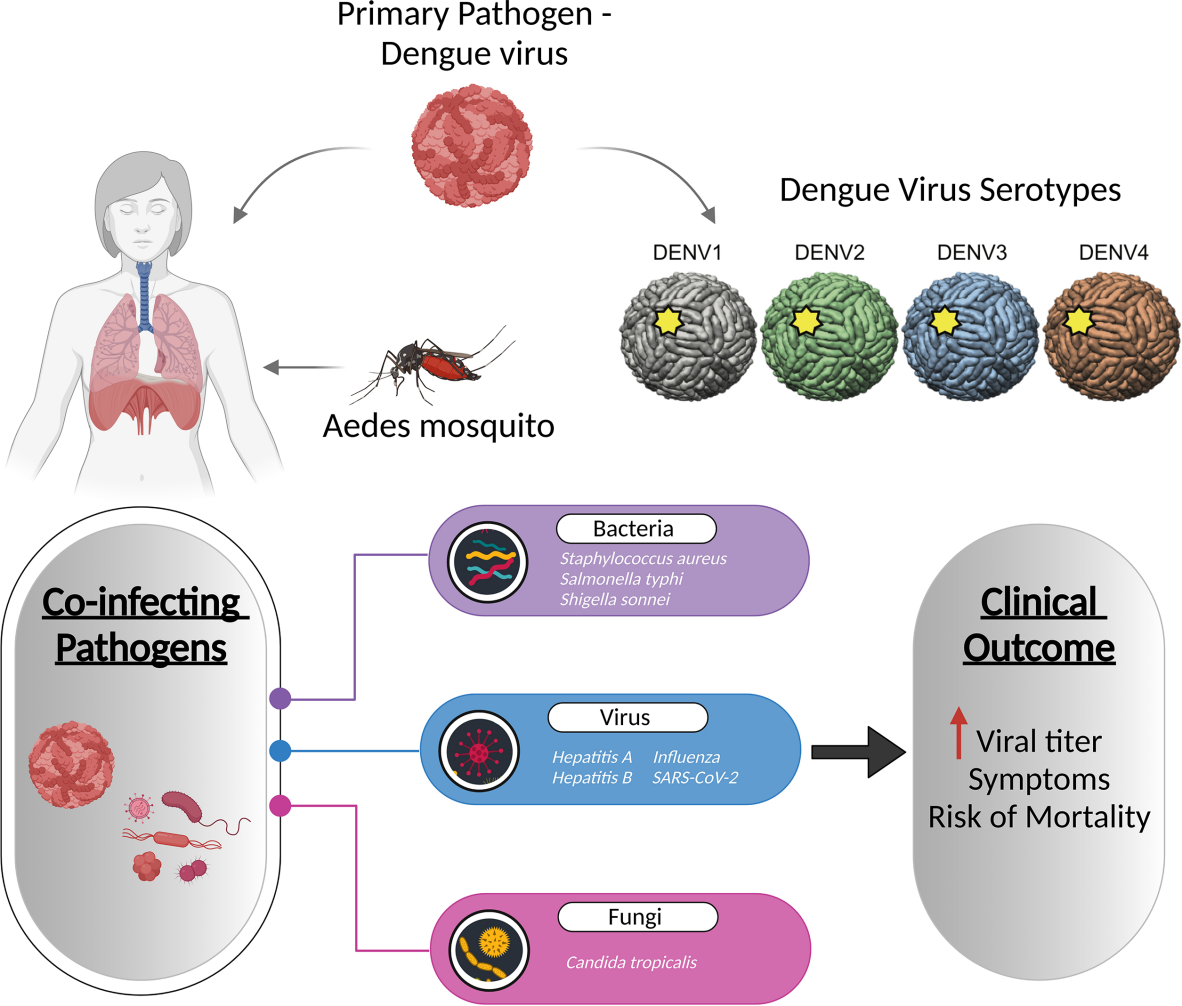
Co-infection of dengue with viruses, bacteria, and fungi increases the dengue viral titre, and even increases the symptoms and mortality of the disease. The figure depicts the known pathogens co-infecting along with the dengue virus and their association with disease severity.

Dengue, caused by the four dengue virus serotypes (DENV 1–4), is the most prevalent and rapidly spreading mosquito-borne viral disease of human beings, resulting in an increased frequency of epidemics and severe dengue disease (122). Co-infections with bacterial, viral, and fungal species are common in dengue patients, with bacterial co-infections being the most common. According to case reports by Mattia Trunfio et al., bacterial co-infections in dengue patients have been linked to sequelae like pneumonia and prolonged fever, as well as an increased death risk (123). Although limited, concurrent *Staphylococcus aureus* infection with dengue has been reported to cause pneumonia, exacerbating dengue symptoms (124). *Salmonella typhi*, causing typhoid fever, has also been linked to dengue, with the co-infection rate of 7.8% (125). A case study revealed that a patient with a chronic fever and diarrhoea had co-infections with dengue and *Shigella sonnei* (126). Fungal infection by *Candida tropicalis* has also been diagnosed with dengue (127). Viral co-infections include hepatitis A, hepatitis B, chikungunya virus, influenza virus, and SARS-CoV-2 that results in increased dengue virus titers (**Figure 4**) (128–132).

Despite the fact that an estimated 100-400 million infections occurring each year, with no specific treatment for the disease, and the availability of only a few reports regarding the co-presence of bacterial isolates, till date, not even a single metatranscriptome study has attempted to identify the functional role of TAMs in dengue (133). The link between DENV infection and concurrent bacteremia highlights the importance of focussed studies on identifying the active microbial community. While there have been reports of co-infections with primary Dengue infection, most of these discoveries have been serendipitous. The need of the hour is to embark on studies to understand, elucidate and explain the causal link with the observed disease severity sub-phenotypes and clinical outcome. It is even more relevant for tropical and subtropical countries, especially as we are living in a connected world with the possibility of global health concern.

## Looking ahead: gaps, needs and challenges

Some enduring questions like, why certain hosts develop the disease while others remain healthy? Why do some patients experience severe and potentially fatal infections, while others remain asymptomatic with the same infecting pathogen? They remain unanswered, even after years of the emergence of infectious diseases. What causes these various disease sub-phenotypes merits focused and sustained attention for future pandemic preparedness, taking forward the learnings from the COVID-19. Beyond understanding the underlying immune response to the disease, and the fact that more than 97% of infected patients tend to survive the infection, identifying the major modulators are essential for public health decision making. As emphasized in the review, the human microbiota play an indispensable role in maintaining the host homeostasis *via* different metabolites and their interactions as well as acting as a reservoir for the opportunistic pathogens to express under favourable conditions, which needs to be further investigated.

In recent times, probiotics have drawn a lot of interest since the microbial dysbiosis is linked to an increased risk of infection. These are the essential dietary components that combine the live beneficial microorganisms that are important in restoring gut microbiota health (134). They have been successfully used as microbial-based therapeutics to fight against infectious pathogens and in turn, reducing the risk of infectious diseases. As gut and lung microbiomes play a significant role in modulating the diseases caused by the SARS-CoV-2 and influenza virus, there have been ongoing clinical trials for using probiotics in an effort to develop prophylactic and therapeutic strategies. Brahma et al. summarized the clinical trials studies that investigates the potential therapeutic role of probiotics for COVID-19, including *Lactobacillus Coryniformis K8*, *Lactobacillus rhamnosus*, *Lactobacillus reuteri*, *Bifidobacterium longum*, *Bifidobacterium animalis subsp. Lactis*, and *Pediococcus acidilactici* (135). Many other studies conducted during the COVID-19 pandemic reported a significant improvement in the symptoms of the patients receiving probiotics‐assisted therapy, along with a reduced occurrence of respiratory tract infection by establishing the oropharyngeal microflora (136, 137). Probiotics have been shown to inhibit influenza virus infection in several mice studies. For e.g. intranasal administration of *Bifidobacterium longum* provides protection by lowering the levels of inflammatory cytokine (including IL-6) and type 1 and 2 interferon, as well as increasing levels of interferon-λ and surfactant protein D (138), while another study showed the protective effects of *Lactobacillus mucosae* and *Bifidobacterium breve* in regulating the immune responses and improving the clinical symptoms (139). Additionally, probiotics have been shown to provide health benefit by enriching the antibody responses to influenza vaccination. Studies have demonstrated that daily intake of probiotics supplements improves the vaccine efficacy (140, 141).

**Figure 5:**
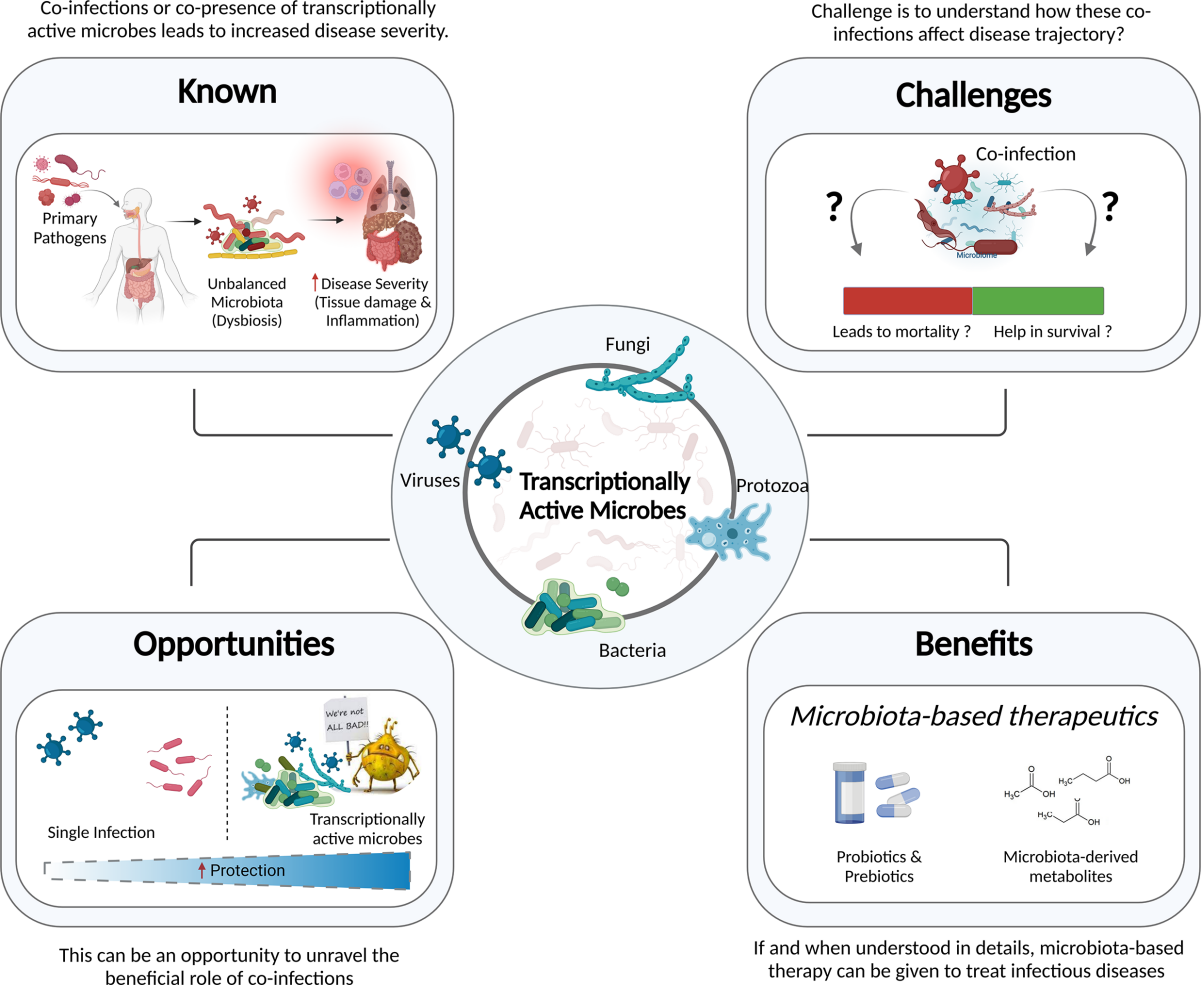
Information to Knowledge. Understanding the known, overcoming the challenges, transforming them into opportunities, and reaping the benefits of this knowledge - in public health and clinical decision making - will augment future pandemic preparedness.

With the recent advances in the molecular biology tools and NGS, our understanding of the dynamic configuration of the human microbiota has grown exponentially. For those microorganisms that are transcriptionally active, the application of RNA-based detection techniques can help with precise taxonomic identification of the microbial species involved in a disease, regulating the consequences. Recent work on TAMs, is one of many important, ongoing efforts to realize the microbiome’s significant translational potential in disease elucidation. As highlighted in this review, the importance of TAMs in addition to total microbiota requires a detailed investigation to determine which microbial communities or specific microorganisms are truly active, which is still lacking. The primary pathogen and the host response to infection may affect the microbial community structure, causing these TAMs to co-infect alongside the primary pathogen, resulting in either protection or a more severe disease outcome (**Figure 5**).

The host’s lifestyle and nutritional habits also have a significant impact on the composition and function of the microbiome (142). During COVID-19, preventive measures, such as mask usage, hand hygiene, and social distancing were implemented in order to combat the disease. Although these strategies reduced the transmission of the SARS-CoV-2, it had a great impact on the human microbiota as well. Studies highlight that the prolonged use of face masks resulted in the alteration of the healthy skin microbiome, making skin more susceptible to fungal and yeast infections, candidiasis and malassezia (143). Additionally, bacterial species, *Staphylococcus epidermidis*, *Staphylococcus aureus*, and *Bacillus cereus* were abundant on the face-side of the mask (144). However, it is currently unclear in absence of specific studies, if this finding of microorganisms on the face masks is in any way affecting the remodelling of the TAMs.

While hand hygiene products are effective against COVID-19, it has also been documented that the excessive and continuous use of hand sanitizers is related to gut dysbiosis. Exposure to triclosan (TCS), an antibacterial chemical found in various hand sanitizers, toothpastes, cookware, and clothes disturbed the gut microbiome in C57BL/6 mice (145). It has also been established that by adversely harming the beneficial microorganisms, prolonged and direct exposure to chemicals found in hand sanitizers may further compromise the integrity and functions of the skin (146).

The co-infection dynamics can be greatly understood utilizing different model systems, such as cell lines, animal models, organoids, as well as mathematical models (19). Several studies have used these models to potentially explain the role of co-infections in modulating disease outcomes; for e.g., Bao et al. discovered increased mortality and pulmonary damage in the SARS-CoV-2 and H1N1 co-infected mice as well as the ferret model (147). Establishing the appropriate models for investigating the co-infections might aid in taking the field forward. It would be fascinating to examine co-infections in 3D organoid cultures, such as the development of lung organoids to study bacterial co-infection in patients with respiratory illness, like COVID-19. Further research in this field is imperative to uncover more details regarding the delicate yet regulatory role underlying the mechanisms of the host, pathogens and active microbiome interactions in infectious diseases.

## Author contributions

AY has read the relevant literature, synthesized the findings, wrote the manuscript and made the figures. RP planned the study, wrote the manuscript, coordinated the manuscript outline, figures and future challenges and opportunities. All authors contributed to the article and approved the submitted version.

## Funding

The study was supported by Bill and Melinda Gates Foundation (BMGF), INV-033578 and INV-030592.

## Acknowledgments

Authors acknowledge Partha Chattopadhyay for proofreading the manuscript and suggestions for improvement. AY acknowledges the CSIR for her Research Fellowship.

## Conflict of interest

The authors declare that the research was conducted in the absence of any commercial or financial relationships that could be construed as a potential conflict of interest.

## Publisher’s note

All claims expressed in this article are solely those of the authors and do not necessarily represent those of their affiliated organizations, or those of the publisher, the editors and the reviewers. Any product that may be evaluated in this article, or claim that may be made by its manufacturer, is not guaranteed or endorsed by the publisher.
